# An Adaptive Track Segmentation Algorithm for a Railway Intrusion Detection System

**DOI:** 10.3390/s19112594

**Published:** 2019-06-06

**Authors:** Yang Wang, Liqiang Zhu, Zujun Yu, Baoqing Guo

**Affiliations:** 1 School of Mechanical, Electronic and Control Engineering, Beijing Jiaotong University, Beijing 100044, China; 12116331@bjtu.edu.cn (Y.W.); zjyu@bjtu.edu.cn (Z.Y.); bqguo@bjtu.edu.cn (B.G.); 2 Key Laboratory of Vehicle Advanced Manufacturing, Measuring and Control Technology (Beijing Jiaotong University), Ministry of Education, Beijing 100044, China

**Keywords:** railway intrusion detection, scene segmentation, scene recognition, adaptive feature extractor, convolutional neural networks

## Abstract

Video surveillance-based intrusion detection has been widely used in modern railway systems. Objects inside the alarm region, or the track area, can be detected by image processing algorithms. With the increasing number of surveillance cameras, manual labeling of alarm regions for each camera has become time-consuming and is sometimes not feasible at all, especially for pan-tilt-zoom (PTZ) cameras which may change their monitoring area at any time. To automatically label the track area for all cameras, video surveillance system requires an accurate track segmentation algorithm with small memory footprint and short inference delay. In this paper, we propose an adaptive segmentation algorithm to delineate the boundary of the track area with very light computation burden. The proposed algorithm includes three steps. Firstly, the image is segmented into fragmented regions. To reduce the redundant calculation in the evaluation of the boundary weight for generating the fragmented regions, an optimal set of Gaussian kernels with adaptive directions for each specific scene is calculated using Hough transformation. Secondly, the fragmented regions are combined into local areas by using a new clustering rule, based on the region’s boundary weight and size. Finally, a classification network is used to recognize the track area among all local areas. To achieve a fast and accurate classification, a simplified CNN network is designed by using pre-trained convolution kernels and a loss function that can enhance the diversity of the feature maps. Experimental results show that the proposed method finds an effective balance between the segmentation precision, calculation time, and hardware cost of the system.

## 1. Introduction

With a continuous increase in the public’s expectation for railway safety, railway intrusion detection systems require more effective technology to detect objects intruding into the track area and to provide real-time alarm information for the command center [1]. Railway intrusion behavior is defined as an object intruding into the track area and endangering the safe operation of trains. Typical intruding objects include rocks falling from a hill beside railway line or a tunnel entrance, pedestrians, vehiclesand their cargo staying in the railroad crossing area or falling from the bridge over the railway. 

**Figure 1:**
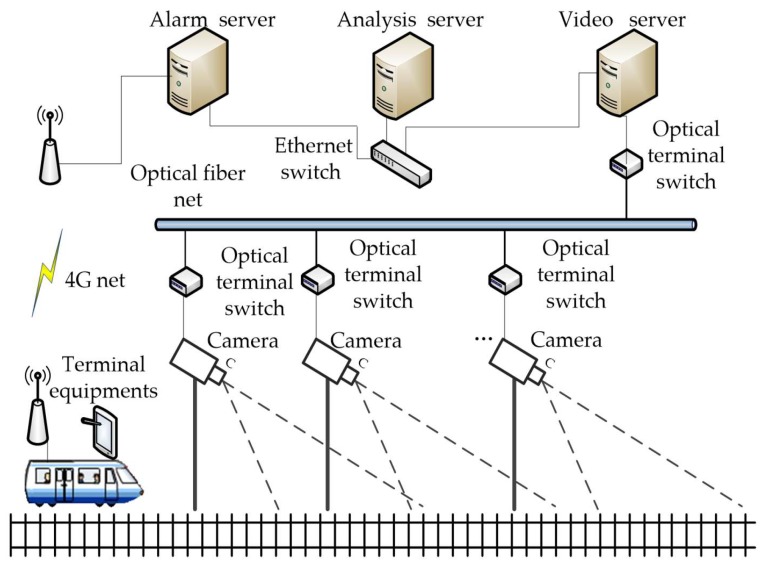
Structure of the railway intrusion detection system.

Depending on the detecting principle, railway intrusion detection systems can be divided into two categories: the contact type and the non-contact type. A representative of the contact type is the protective metal net installed along the line to block an object from intruding into the clearance, and the system will send the alarm information when the physical deformation of the net is measured by a dual-power sensor [2] or fiber grating sensor [3,4]. The systems based on the non-contact measurement technology use infrared sensor [5] or laser scanner [6,7] to get the size and location of the intruding object [8]. Video surveillance is also widely used as another kind of non-contact intrusion detection systems because of the large monitoring area, convenient installation, maintenance, and good observable results [9]. As shown in Figure 1, we established an intrusion detection system for the Shanghai–Hangzhou high-speed railway in China. The system contains data process servers, communication networks, and 1550 cameras, including both of fixed and PTZ cameras.

**Figure 2:**
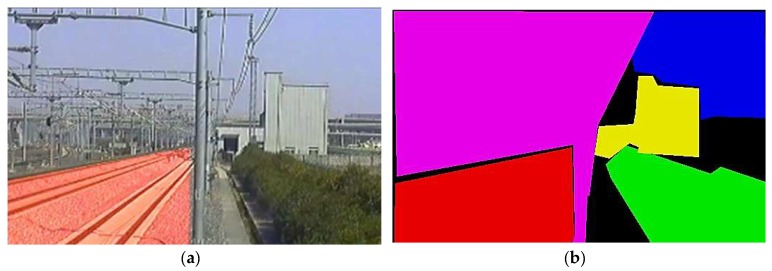
Railway scene and the local areas, labeled manually. The image quality is susceptible to external influences, such as the illumination, weather, and even the dust on the lens. (**a**) The red area is the track area to be surveilled. The track area includes the rails, sleepers, subgrades, or high-speed railway slabs. (**b**) Labeling the different area of the railway scene with different colors by manual, including track area (**red**), sky (**blue**), catenary system (**purple**), green belt (**green**), and ancillary buildings (**yellow**). The precision depends on the patience of the manual operator.

The threat level of an intrusion behavior will be evaluated by the category, location, and moving trajectory of the object with respect to the track area. The information of the intruding object can be extracted by image processing algorithms, e.g., density-based spatial clustering of applications with noise (DBSCAN) [10], fast background subtraction (FBS) [11], Kalman filtering [12], principal components analysis (PCA) [13]. DBSCAN uses extremum points of scan sequence as core objects of clustering, and the movement and distribution characters are used to judge whether the cluster is a train or other foreground object. FBS projects the scene image into one dimension (x or y dimension) to locate position of the foreground object by the change of the peak value. KF classifies the objects acquired via image background subtraction by support vector machine (SVM), and then using the Kalman-filter tracking algorithm to analyze the behavior and moving trend of the objects. PCA projects the statistic of the scene images and the successive images in a transformation space and calculates the Euclidean distance, which is greater than a threshold, is considered like belonging to motion objects. Most of the above-mentioned algorithms only focus on the foreground object, rather than the track area in the background. Therefore, the position and boundary of track area are still delineated manually in advance, as shown in Figure 2. 

The precision of the track area boundary directly affects the reliability of intrusion detection. With an increasing number of surveillance cameras along the railway line, especially as some PTZ cameras will change their focal lengths and angles temporarily for different applications, manual labeling has become time-consuming and laborious. Thus, for the efficiency of the railway intrusion detection system, a scene segmentation algorithm is needed to recognize the track area and delineate the boundary automatically. The algorithm will be applied to initialize surveillance areas after the installation of all cameras, and to relearn them when the operator adjusts PTZ cameras. Meanwhile, the practical engineering application has many requirements: the relevant image parsing algorithm should not only have good segmentation precision and classification accuracy, but also be able to process temporarily changing scenes quickly. In addition, the algorithm should have small number of parameters and can be easily applied into the data processing servers with different hardware configurations and even into the embedded surveillance equipment in the field.

Currently, there are two ways to parse a scene. The traditional way will segment the scene image into superpixels, ultrametric contour maps (UCM), or other fragmented segment regions [14,15], and then combine them into candidates of objects or local areas based on Markov random fields (MRFs), conditional random fields (CRFs), multiscale combinatorial grouping (MCG), or other rules [16,17,18]. These traditional methods will generate fragmented regions with precise boundaries and require time-consuming iterative calculations to form a best candidate of an object or a local area. In addition, category information of objects cannot be produced. The second way relies on deep neural networks, e.g., fully convolutional networks (FCN) [19,20], to process the feature extraction, combination, segmentation, and recognition at the same time. A FCN can achieve the segmentation and recognition in a single process. One drawback of FCNs is that the boundary line generated is usually a smooth curve, which will miss the corner of the track area. In addition, FCN has big memory footprint and needs a GPU to accelerate its large amount of computation.

In this paper, we propose an adaptive segmentation algorithm that can take advantage of both methods while avoiding their shortcomings. Like the existing traditional methods, we extract the texture distribution of the image to generate the boundary point with different weight for segmenting the image into small fragmented regions, and then the regions are combined into local areas with precise boundaries; finally, we apply a specially designed convolutional neural network (CNN) for the area’s classification without the need of GPU. Our main contributions include:

- To accelerate the generation of small fragmented regions, we propose a method to find the optimal set of Gaussian kernels with adaptive directions for each specific scene. By making full use of the straight-line characters of the railway scene, a smaller number of adaptive directions are calculated according to the maximum points in Hough transformation rather than being chosen from a set of fixed angles in the traditional way. As a result, the calculation time for the boundary extraction and fragmented region generation is cut in half;
- A new clustering rule based on the boundary weight and the size of the region is set up to accelerate the combination of the regions into local areas. The number of regions is reduced in the process of weak boundary point removal by filtration, and the smallest remaining region is combined with its neighbor region, which shares the weakest boundary;
- We propose a specially designed CNN model to achieve the fast classification of local areas without the need of GPU. The local areas are divided into two categories: the track area which is used to judge the intrusion behavior and the rest area which is unrelated to the intrusion. The convolution kernels are pre-trained, and a sparsity penalty term is added into the loss function to enhance the diversity of the convolutional feature maps.

The rest of this paper is organized as follows. In Section 2, we review the related works on image parsing algorithms. Section 3 explains the proposed fast image segmentation process. Section 4 explains the proposed simplified CNN network structure and the optimization process. Section 5 presents the experimental results and discusses them. The last section summarizes our conclusions.

## 2. Related Work

### 2.1. Image Parsing by Traditional Methods

To segment an image using the traditional methods, the first step is to calculate the correlation between the adjacent pixels in the scene image, and then segment the image into fragmented regions by a certain convergence criterion [21,22]. The superpixel algorithm, for example, converts the image from the RGB color space to CIE-Lab color space to form a five-dimensional vector (brightness, color A, color B, and position x, position y), and the vector distance between two pixels representing their similarity, is used to generate the small segments patches [14,23]. A spatial pyramid descriptor fuses the gray, colored and edge gradient into one feature vector for the SVM classifier to recognize a traffic sign [24]. The image can also be converted into the YCbCr color space, and the local texture features in different channels are matched with the artificially designed template to locate the position of the traffic sign [25]. Therefore, converting the image from the RGB color space into another feature space can obtain more dimensional information channels: brightness, texture, and other feature maps besides RGB color. 

To achieve the final segmentation, the fragmented regions need to be combined. The internal correlations among the adjacent regions are calculated according to different rules, and the regions are combined into local areas according to their correlation values. For example, the K-means clustering rules are used in different practical engineering applications, such as object detection for the synthetic aperture radar (SAR) image and the sea scene [26,27,28]. The MCG algorithm is another grouping strategy using random forests to combine the multiscale regions into highly accurate object candidates.

MCG can process one image (pixel size 90 × 150) in 7 s, and the mean Intersection over Union (IU) is about 80% [18,29,30]. The clustering rules influence the combination precision, which is also directly proportional to the calculation time; as a result, MCG is suitable for the initial or post processing of a fixed scene, not for real-time processing of temporarily changing scenes. Therefore, to accelerate the whole scene segmentation process, we choose to improve the traditional methods in both generation and combination of the fragmented regions while maintaining the segmentation precision.

### 2.2. Image Parsing by Deep Learning Methods

Deep learning methods have also been widely used in image parsing recently, e.g., various convolutional networks, which have better robustness to image translation, rotation, scaling, and distortion. Deep learning methods can be divided into three types: image classification [19], object detection [31], and pixelwise prediction [20]; and the complexity of their network structures increases from image-wise to pixel-wise. 

For the pixelwise segmentation of a scene, the convolutional networks can be combined with the superpixels, the random effect model and the texture segmentation to generate the pixelwise labels [32], and also can be used as a classifier to classify the feature maps containing RGB and depth information [33,34,35]. FCN can even process feature extraction, combination, segmentation, and recognition at the same time, also achieving a pixelwise prediction [20]. 

Depending on the details of different FCN structures, the mean IU of FCN is about 80%, the accuracy is about 90%, and the quantity of the parameter is about 57 M to 134 M. The massive number of parameters and computation need a GPU with big memory to handle the operation, leading to a high cost for practical applications. Therefore, we choose to use the traditional methods to get the precise boundary of the local area first, and then use a simplified CNN only to classify the local areas without the need of GPU. However, the reduction of the network size causes low accuracy in the classification, so extra care has to be taken in optimizing the network structure and the training process.

## 3. Railway Scene Segmentation

As shown in Figure 2b, typical railway scene consists of different areas, including track area, sky, catenary system, green belt, and ancillary buildings. The precision of the track area boundary directly affects the reliability of the judgement about whether the intrusion occurs or not. The track area is defined as the clearance area including rails, sleepers, subgrades or high-speed railway slabs, as shown in Figure 2a. To avoid manual labeling, a fast and precise railway scene segmentation algorithm is proposed. 

**Figure 3:**
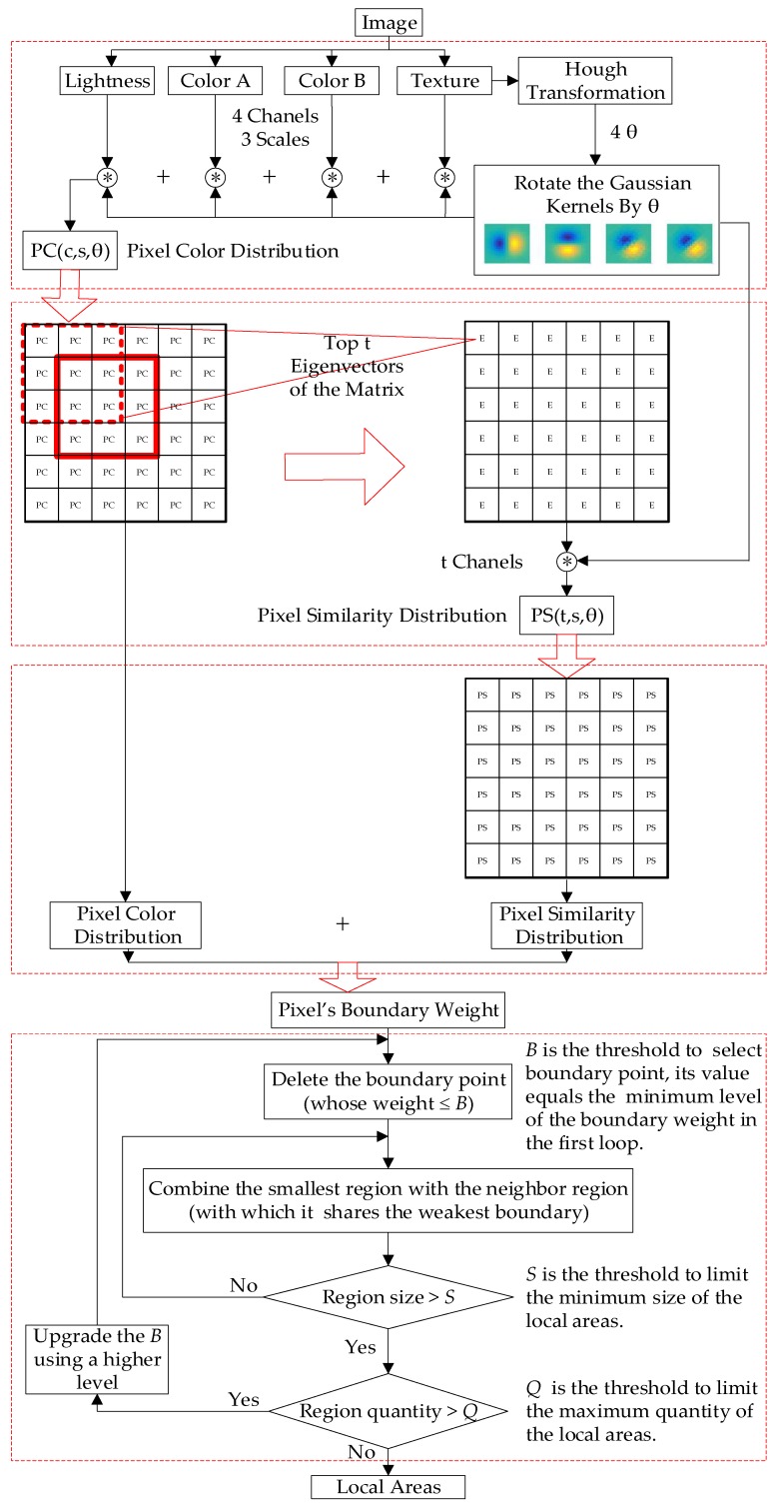
The procedure to segment an image into fragmented regions and combine them into local areas.

Figure 3 illustrates the outline of the proposed algorithm. We first calculate the feature distribution in a small image patch (pixel size 15×15) representing the central pixel of the patch, then evaluate the central pixel’s probability of being a boundary point, and finally use the boundary weights to segment the image according to a fast combination rule. Unlike the traditional method, we use a smaller set of adaptive Gaussian kernels to extract the pixel color (*PC*) distribution and pixel similarity (*PS*) distribution of the image in different channels *C* and by different scales *S*. The Gaussian kernels are rotated by a set of adaptive θs, calculated from Hough transformation. The detailed procedure of boundary weight generation is described in the remainder of this section.

### 3.1. Generation of Fragmented Regions

Firstly, we convert the image into the CIE lab color space, getting 3 channels: brightness, color A, and color B. Images in different channels will be scaled by s=(0.5,1,2). In each channel, the image is convoluted with Gaussian kernels to get the color value distribution; each kernel has a special orientation angle θ. Define G(x,y,θ,c,s) as the convolution result at pixel P(x,y), with angle θ, in channel c, by scale s. Then PC, the pixel’s color distribution, can be obtained by

(1) $$PC(x,y,\theta )=\sum_{s}\sum_{c}\alpha_{c,s}G(x,y,\theta ,c,s)$$

where $\alpha_{c,s}$ is a weighting coefficient.

Secondly, define $S_{imilarity}(i,j)$ as the maximum PC value of all pixels on the line $l_{i,j}$ connecting two pixels i and j in an small image patch by Equation (2), representing the similarity between pixel i and j.

(2) $$S_{imilarity}(i,j)=\exp(-Max\left\{PC(x,y)|(x,y)\in l_{i,j}\right\})$$

Calculate the similarity of each pixel $i_{x,y}$ in the patch and the central pixel $j_{center}$, assign $S_{imilarity}(i_{x,y},j_{center})$ to each element MS(x,y) of the Matrix of Similarity MS, and assemble MS representing the similarity matrix between each pixel in the image patch and the central pixel.

Calculate the top t eigenvalues and eigenvectors of MS. Assign the eigenvector to the central pixel P(x,y) marked as e(x,y,t), forming a feature map E of the image, representing the similarity of the adjacent points. Again, in each dimension of the feature map, convolute E(t) with Gaussian kernel of orientation θ to get the similarity distribution. Define g(x,y,θ,t,S) as the convolutional result at location E(x,y), with angle θ, in dimension t, by scale s. Then, the pixel’s similarity value distribution can be obtained as

(3) $$PS(x,y,\theta )=\sum_{s}\sum_{t}\beta_{c,s}g(x,y,\theta ,t,s)$$

where $\beta_{c,s}$ is a weighting coefficient.

Finally, B(x,y), the possibility of the pixel P(x,y) being a boundary point, can be estimated by

(4) $$B(x,y)=\sum_{\theta }PC(x,y,\theta )+\sum_{\theta }PS(x,y,\theta )$$

### 3.2. Finding the Optimal Set of Gaussian Kernels

It can be found that, in the process of estimating B(x,y), convolution operations (Equations (1) and (3)) using Gaussian kernels with different orientation angles θ cost most of the computation, which can be reduced if a smaller set of Gaussian kernels are used. The traditional UCM algorithms choose fixed size of $\theta =(\theta_{1},\theta_{2},\theta_{3}...)$ with 8 or 16 values uniformly distributed from 0 to π. Here we propose to utilize the characteristics of the railway scene to find a much smaller set of useful orientation angles and thus a smaller set of Gaussian kernels. Usually, in railway scene, there is a clear vanishing point (VP), and the boundaries of many local areas are lines passing through the VP. Therefore, if we can automatically adjust the candidate θ for each specific scene to enhance the weights of the line boundary points of the relevant areas, then we will be able to use a smaller set of θ to accelerate the process.

We propose to find the candidate θ by filtering the original image with a Canny kernel [36], and then convert the obtained texture feature into the Hough coordinate system using

(5) $$\rho =x\cos\theta^{\prime }+y\sin\theta^{\prime },-\frac{\pi }{2}<\theta^{\prime }<\frac{\pi }{2}$$

**Figure 4:**
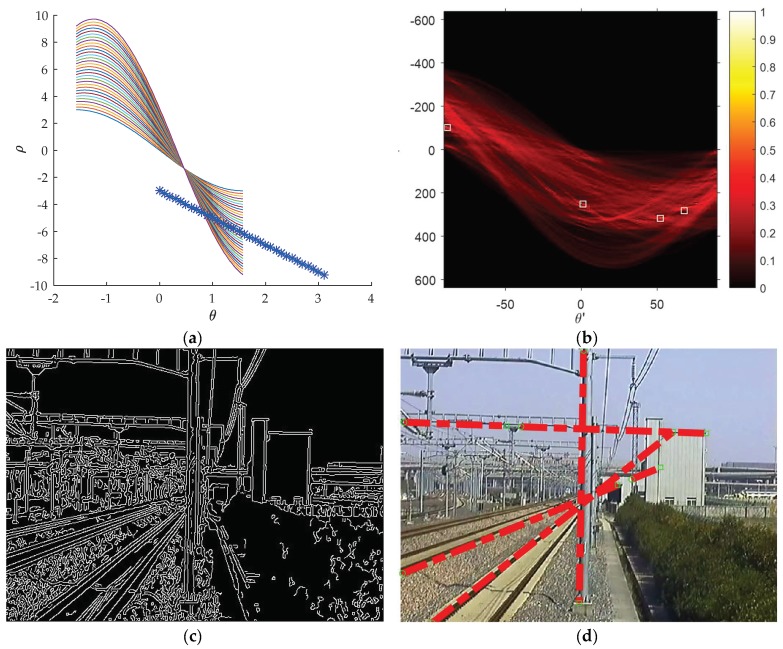
Using Hough transformation to detect the most significant lines in the Hough coordinate system. (**a**) The intersection point of a group of curves in the Hough coordinate system means there are a group of collinear points in the Cartesian coordinate system. (**b**) The more the curves intersect in the Hough coordinate system, the lighter the intersection point is, meaning that there are more collinear points along this line in the Cartesian coordinate system. (**c**) The texture feature maps filtered by canny filter. (**d**) The top four significant lines.

As shown in Figure 4a, each curve in the Hough coordinate system stands for one point in the Cartesian coordinate system. If the curves (colorful curve lines in Figure 4a) have one intersection point in the Hough coordinate system, then the corresponding points (blue point in Figure 4a) in the Cartesian coordinate system are collinear. 

Let $H\left(\theta^{\prime },\rho \right)$ be the number of curves intersecting at point $\left(\theta^{\prime },\rho \right)$ and find the point with maximum $H\left(\theta^{\prime },\rho \right)$, where there are the largest number of points which are collinear on the corresponding line in the Cartesian coordinate system. The line can be expressed as

(6) $$y=-\frac{1}{\tan\theta^{\prime }}x+\frac{\rho }{\sin\theta^{\prime }}=kx+b$$

**Figure 5:**
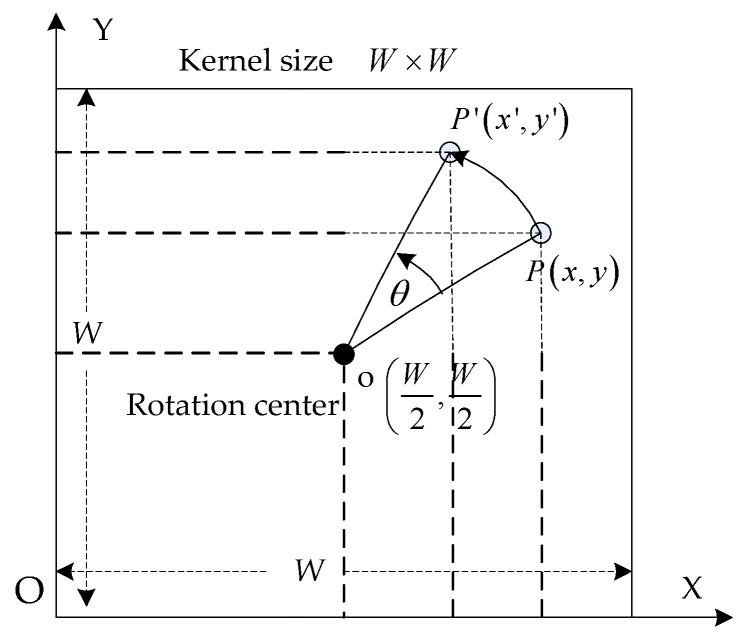
Calculating the rotation matrix of the Gaussian kernel. The rotation center is on the kernel center.

To find a small set of four orientation angles, one can take the top four maximum θ in $H\left(\theta^{\prime },\rho \right)$, e.g., the points with highest ‘lightness’ in Figure 4b: $\theta^{\prime }=$ 68°, 52°, 0° and −88°. Here we change the $\theta =90{}^{\circ}-\theta^{\prime }=$ 22°, 38°, 90°, and 178° in order to obtain a range of values from 0° to 180° (0–π). Based on the selected set of orientation angles, the Gaussian kernels can be constructed correspondingly by rotating the Gaussian. As shown in Figure 5, in the Cartesian coordinate system X-O-Y, point P(x,y) rotates around the point $o(\frac{W}{2},\frac{W}{2})$ an angle θ to $P^{\prime }(x^{\prime },y^{\prime })$, which can be formulated as

(7) $$\begin{array}{l} \left[\begin{array}{ccc} x & y & 1 \end{array}\right]=\left[\begin{array}{ccc} x^{\prime } & y^{\prime } & 1 \end{array}\right]\left[\begin{array}{ccc} 1 & 0 & 0\\ 0 & -1 & 0\\ -0.5W & 0.5W & 1 \end{array}\right]\left[\begin{array}{ccc} \cos\theta & -\sin\theta & 0\\ \sin\theta & \cos\theta & 0\\ 0 & 0 & 1 \end{array}\right]\left[\begin{array}{ccc} 1 & 0 & 0\\ 0 & -1 & 0\\ 0.5W & 0.5W & 1 \end{array}\right]\\ =\left[\begin{array}{ccc} x^{\prime } & y^{\prime } & 1 \end{array}\right]\left[\begin{array}{ccc} \cos\theta & \sin\theta & 0\\ -\sin\theta & \cos\theta & 0\\ -0.5W(\cos\theta -\sin\theta -1) & -0.5W(\sin\theta +\cos\theta -1) & 1 \end{array}\right] \end{array}$$

**Figure 6:**
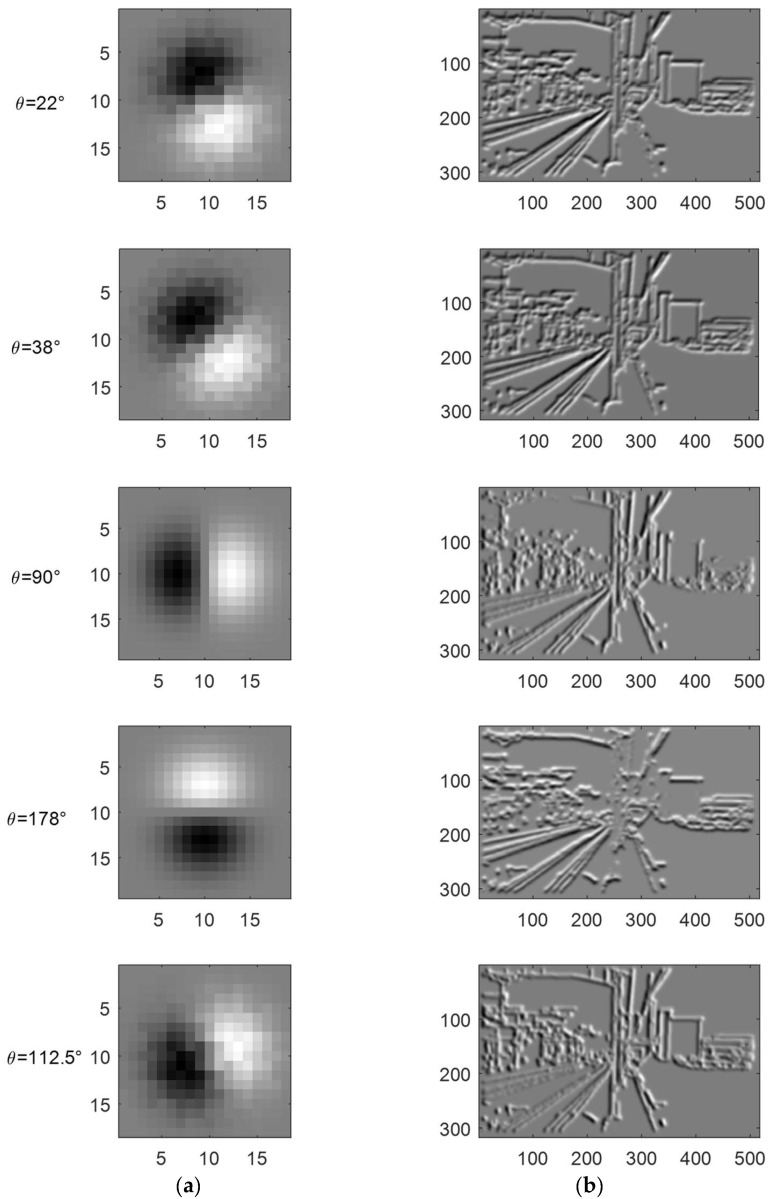
Different kernels and the convolution results of CIE-lab color L channel. (**a**) First order derivative Gaussian kernels rotated by five angles. (**b**) Results of the Gaussian convolution.

Figure 6 shows several Gaussian kernels rotated by the optimal set of θ=22°, 38°, 90°, and 178° obtained above and θ= 112.5°, one of the eight uniformly-distributed values commonly used in traditional UCM algorithms, respectively. The results show that the features of the horizontal catenary bracket, the vertical catenary column and the declining track are strengthened obviously in the first four filters, contrasting with the feature extraction equality in the fifth filter. The universality of using 8 or 16 uniform values in different angle θ causes a redundant calculation when applied to the railway scene. Therefore, adjusting a smaller number of θ adaptively to filter the feature map can accelerate the boundary weighting to generate the fragmented regions.

### 3.3. Combination Rule

**Figure 7:**
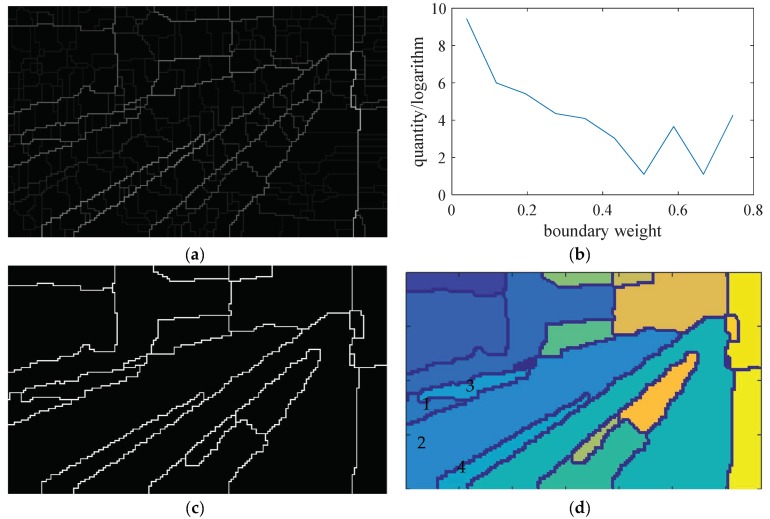

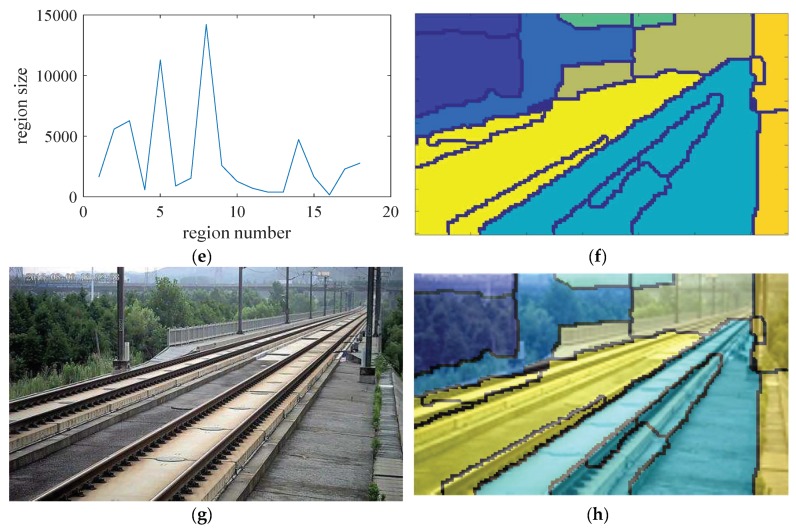
The procedures of combining the fragmented regions into local areas. According to the adjustment and experiments, for the railway scene, the scene image is set to a pixel size of 90 × 150, the number of adaptive θ is reduced to 4, the number of reserved areas *Q* is set to 10, and the smallest fragmented area *S* is set to 10% of the total size of the image. (**a**) Boundary with weight. (**b**) Distribution of boundary weight and quantity. (**c**) Delete the weak boundary. (**d**) Fragmented regions. (**e**) Distribution of the region size and serial number. (**f**) Local areas after the fragmented regions are combined. (**g**) The original railway scene image, (**h**) is the result.

The fragmented regions generated by the adaptive boundary detection are shown in Figure 7a. The higher the boundary weight is, the brighter the point is shown in the gray feature map, indicating that the point is more likely to become a boundary point.

A clustering rule based on both of the boundary weight and the region size is proposed to combine the fragmented regions into local areas. The number of the regions will be reduced in the process of weak boundary point removal by filtration. The smallest remaining region will be combined with its neighbor region, with which it shares the weakest boundary. Repeat this iteration until the statistical parameters meet the requirements. The process is as follows:

1. Let B(m) be the normalized value of the boundary point’s weight $B(x_{m},y_{m})$, where m=1,2,3...M, and Mis the total number of boundary points:
  (8) $$B(m)=sigmoid(B(x_{m},y_{m}))=\frac{1}{1+e^{-B(x_{m},y_{m})}}$$
2. The statistical distribution of the boundary point weight B(i) is shown in Figure 7b. There are many levels of boundary point weights. Choose the minimum level B as the threshold to delete the weak boundary points B(m)≤B;
3. The fragmented regions will be reduced by reconnecting the breakpoints of the boundary line using expansion and corrosion operations, as shown in Figure 7c. The new regions are shown in Figure 7d;
4. The statistical distribution of region size f(n) is shown in Figure 7e, where n=1,2,3...N, and N is the serial number of the regions. Choose the smallest region along its boundary line and find the neighbor region which shares the weakest boundary with it. Then combine them into a new region. As shown in Figure 7d, regions in number 1, 2, 3, and 4 are combined as one new region in Figure 7f;
5. Repeat Step 4 to reduce *N* until the area of the smallest region is larger than a threshold *S*, which is used to limit the minimum area of the remained regions*;*
6. Compare the final *N* with another threshold *Q* to limit the minimum quantity of the remained regions. If N>Q, select the second minimum level weight B and go back to Step 2;

Figure 7g is the original railway scene image, and the Figure 7h is the result of our segmentation algorithm. The railway scene only contains five categories of areas, and the shape of the area is usually in a large and radial pattern. Therefore, we set the minimum area threshold *S* to 10% of the whole image and the maximum quantity threshold *Q* to 10, which will prevent the remained regions from being too fragmented. The remained regions will be adjusted into a standard size of 64 × 64 and RGB 3 channels, after being classified by the CNN in Section 4, the remaining regions with the same labels will be combined as one local area. 

## 4. Local Area Recognition in Railway Scene

To automatically label the local areas in real time without the help a GPU, we design a simplified CNN with less layers and kernels. To compensate the reduced accuracy, the convolution kernels are pre-trained, and a sparsity penalty term is added into the loss function to enhance the diversity of the feature maps.

### 4.1. Structure of Simplified CNN

**Figure 8:**
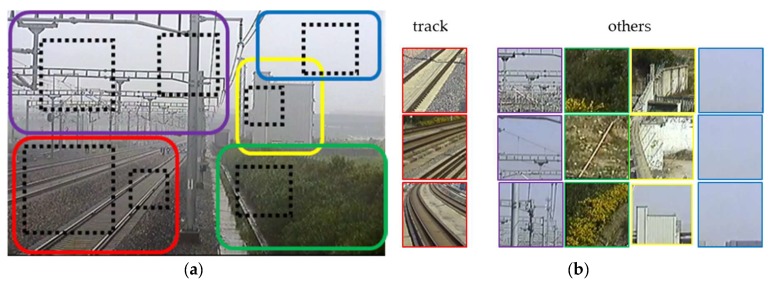
Collecting samples of local areas for CNN training. (**a**) Solid-line rectangles are delineated by manual with labels, including the track area (red), sky (blue), catenary system (purple), green belt (green), ancillary buildings (yellow). The dotted-line boxes are extractor windows. (**b**) The dataset containing two categories for training the CNN.

Before designing and applying a simplified CNN, we first construct a dataset of local area images for training it. As shown in Figure 8, there are mainly five basic categories of elements in a typical railway scene, including track area, sky, catenary system, green belt, and ancillary buildings. To sample the dataset, five solid line rectangles are manually defined to cover the five different areas. We program a simple extraction code to take the image patches using the dotted-line box as samples with the same category of the outer rectangle. We set up a group of constraint parameters to control the dotted box to extract the patches at a random position, by a random scale, maintaining inside of each rectangle. The image patches are adjusted into a pixel size of 64 × 64 and RGB 3 channels to assemble our five-category datasets of railway local area. However, for the specific application of this paper, our target is focused on the track area for judging intrusion behavior, so besides the ‘track’ label, we merge the other four elements into one category labeled as ‘others’. There are 9000 image patches in total, in which 5000 images are used for training our net, 2000 images are used for cross-validation, and 2000 images are used for testing. 

**Figure 9:**
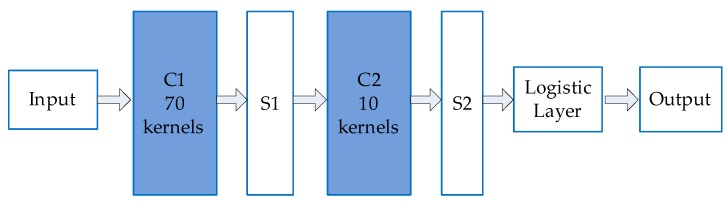
Structure of the simplified CNN. The size of the input image is a pixel size of 64 × 64 with RGB 3 channels. The output is one of the two category labels.

A simplified CNN structure is designed for fast recognition, which consists of an input layer, two convolution layers *C1* and *C2*, two mean pooling layers *S1* and *S2*, and a logistic classification layer, as shown in Figure 9.

As shown in Table 1, we conducted five experiments with different kernel quantities and sizes. It can be seen that increasing the kernel size and quantity may increase the accuracy, but the accuracy is still less than 80%. Although the railway scene is very simple, only containing several typical area categories, the shapes, color, and texture features of the area belonging to the same category are still very complex and different. Therefore, the training process must be optimized to increase the accuracy.

### 4.2. Optimization of the Simplified CNN

**Figure 10:**
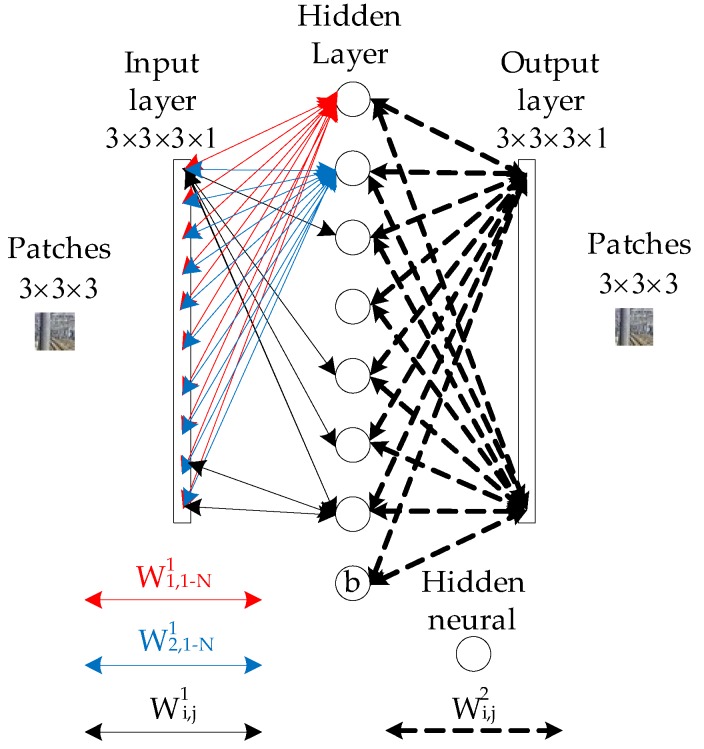
Structure of the autoencoder-decoder network. The hidden layer contains 70 hidden neurons; *W* denotes the weight associated with the connection between neurons; and the network is trained to produce output the same as its input.

**Figure 11:**
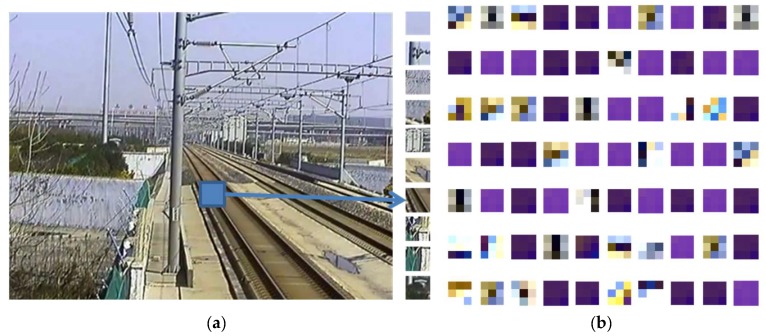
Pre-trained convolution kernels using the autoencoder-decoder algorithm. (**a**) The image patches are extracted from the left railway scene image for the kernel training. (**b**) The pre-trained kernels used in convolution layer *C1*.

To increase the accuracy, kernels are pre-trained to extract better low-level features. The pre-training strategy is based on autoencoder-decoder network; and the $W_{i,3\times 3\times 3}^{1}$ after training in first layer is applied as the convolution kernel in the first convolution layer *C1*, as shown in Figure 10 for the case with kernel size of 3×3 and in RGB 3 channels. During the training, 3×3patches in RGB 3 channels are randomly selected from random railway scene images, as shown in Figure 11a. The result of the pre-trained kernels is shown in Figure 11b, where the patches and the kernels are all in RGB 3 channels. 

After pre-training, the input weights of each neuron in the hidden layer are used as the initial weights of kernels in the first convolution layer *C1* in Figure 9. The rest of CNN in Figure 9 are randomly initialized and then trained by using a backpropagation algorithm (stochastic gradient descent, SGD). To enhance the diversity of the feature maps, a sparsity penalty term is added into the loss function J as

(9) $$J=\left\{\frac{1}{P}\sum_{p=1}^{P}\frac{1}{2}{\left[h\left(e_{p}^{}\right)-l_{p}^{}\right]}^{2}\right\}+\tau \sum_{f=1}^{10}\left[\chi \mathrm{lg}\frac{\chi }{\eta_{f}}+(1-\chi )lg\frac{1-\chi }{1-\eta_{f}^{}}\right]$$

where

(10) $$\eta_{f}=\frac{1}{P}\sum_{p=1}^{P}\sum_{u=1}^{29}\sum_{v=1}^{29}O_{f,e_{p}}^{\left(2\right)}(u,v)$$

$e_{p}$ is the *p*-th input image, $l_{p}$ is the ground truth label, there are totally *P* images in the dataset, $h(e_{p})$ is the output label, τ is the weight of the sparsity penalty term, χ is the sparsity parameter (a smaller value close to 0, e.g., 0.05), $\eta_{f}$ is the average output of the f-th feature map in convolution layer *C2* (averaged over the training dataset), and $O_{{}_{f,e_{p}}}^{\left(2\right)}(u,v)$ is the value at position (u,v) in the *f*-th feature map of the input $e_{p}$ in the second convolutional layer *C2*, the size of the feature map is 29 × 29 pixels. 

In the process of backpropagation, the sparsity penalty item will suppress the average output of all feature maps in the second convolutional layer *C2*, but enforce the output of one feature map at the same time, so as to enhance the diversity of the feature maps and improve the accuracy. The learning rate is set to 0.1, and the decay of the learning rate is 0.001 after each iteration, the final value of *J* should be less than 0.05.

### 4.3. Performance of the Simplified CNN

As shown in Table 2, the accuracies of the simplified CNNs with different structures are all increased by using the proposed optimization method, compared with the results of traditional training method shown in Table 1, e.g., the simplified CNN with 70 kernels (3×3, 3 channels) in *C1* and 10 kernels (3×3, 70 channels) in *C2* is used for the proposed segmentation algorithm. The quantity of the network parameters is only 0.02912M. After the railway scene is segmented and classified, the regions with track labels can be combined together as the final track areas.

## 5. Experiments and Results

### 5.1. Railway Scene Dataset

**Figure 12:**
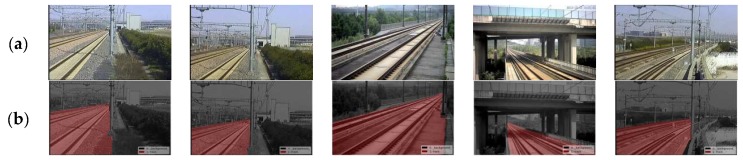
Samples in railway dataset. (**a**) Images from PTZ cameras under different conditions. (**b**) Ground truth of the track area.

We collect images from 16 PTZ cameras at straight lines, curves and bridges in the high-speed railway from Shanghai to Hangzhou, China. For each camera, images are collected from 10 different shooting angles, lenses, and under different illumination conditions from 8:00 a.m. to 5:00 p.m. Examples are shown in Figure 12a. There are totally 1760 scene images in the dataset, in which 1000 images are used in the training dataset, 400 images are used in the cross-validation dataset and 360 images are used in the test dataset. These datasets are used to generate the datasets for our simplified CNN (Section 4.1) and the dataset for training the FCN for the comparison experiments.

### 5.2. Modification of the Workflow for the Case of Small Track Portion

**Figure 13:**
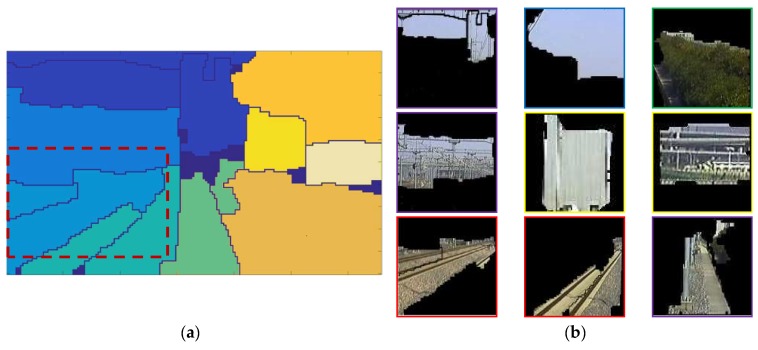

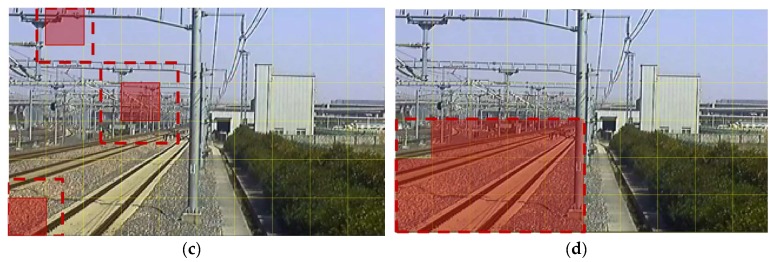
Rough scanning over the scene to find the potential track area. (**a**) Segmentation result of the whole railway scene images. (**b**) Different local areas. (**c**) Scanning the railway scene image roughly using proposed CNN. (**d**) Area in red dotted rectangle is the potential track area, which will reduce segmentation calculation by three-quarters.

For cameras on line sections, track area only takes up a small portion of the scene image, while for the ones at tunnel entrances and bridges over railway line, track area usually takes up most of the scene. As shown in Figure 12b, the red track area takes about 25–70% of the whole scene image for different cameras. That means, the complete-processing workflow (Section 3 and Section 4) would waste a lot of time calculating the boundaries between the ‘others’ areas (Figure 13b) rather than focusing on the potential track area as the red dotted line rectangle shown in Figure 13a. In order to find the potential track area and reduce the segmentation calculation furthermore, we design a partial-scanning workflow to locate the potential position of the track area before the segmentation and classification by scanning over the railway scene roughly using the proposed CNN. As shown in Figure 13c, we firstly divide the railway scene image into 6×10 cells (yellow cell); each cell and its peripheral zone (red dotted line rectangle) are resized to 64×64 pixels, define their classified labels as the representation of its central cell (red area in Figure 13c); the proposed CNN is used to classify these cells and the output labels are used to identify the potential track area roughly as the red area shown in Figure 13d; A minimum enclosing dotted line rectangle is used to adjust the potential track area into a regular shape as shown in Figure 13d. 

The strategy of the partial-scanning workflow reduces the segmentation area, but spends extra scanning time. Thus, the overall processing time depends on the proportion of the track area to the railway scene, as shown in Table 3, the numbers on the left are the scene images in Figure 12, from the left to the right. If the track area takes over more than 88.1% of the railway scene, the performance of the partial-scanning workflow would be worse than the complete-processing workflow. These two workflows can be chosen for different cameras: for those with short focus lens and focus on the near scene full of track area, the complete-processing workflow should be used; for the ones with long focus lens and track area only take a small part of the scene, the partial-scanning workflow should be used.

### 5.3. Metrics

To evaluate the segmentation performance, three criteria are used. The first one is the intersection over union (IU) generally defined as Equation (11), where *L* represents for the ground truth, R represents the segmentation result; the second one is the pixel accuracy (PA) defined in Equation (12) to evaluate the portion of the area which need to be surveilled are segmented; and the extra pixel (EP) as Equation (13) is used to evaluate the portion of segmented areas which do not need to be surveilled. PA would influence the missing part of the track area which would cause a missing alarm, and the EP would influence the extra part of the track area which would cause a false alarm.

(11) $$IU=\frac{L\cap R}{L\cup R}$$

(12) $$PA=\frac{L\cap R}{L}$$

(13) $$EP=\frac{R-L\cap R}{L}$$

### 5.4. Performance of the Proposed Segmentation Algorithm

**Figure 14:**
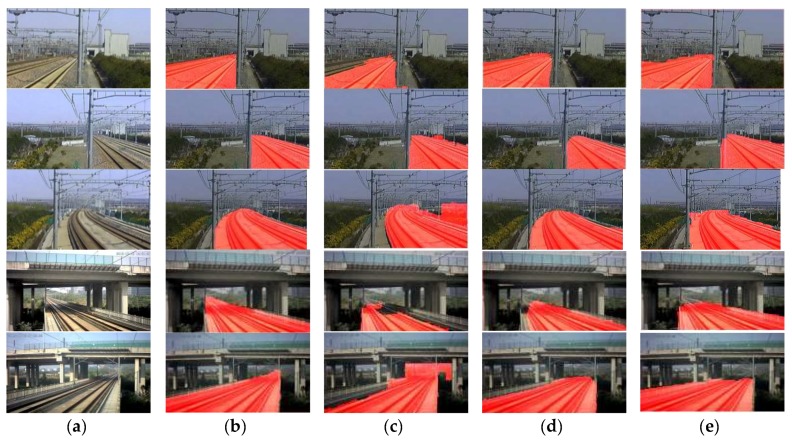
Using different algorithm to detect the track area. (**a**) The original railway scenes. (**b**) Ground truth of track areas. (**c**) Results of the MCG algorithm. (**d**) Results of the FCN algorithm. (**e**) Results of our algorithm.

The proposed algorithm is compared with MCG and FCN using images from railway dataset and some examples are shown in Figure 14. In the experiment, the computation platform is equipped with an Intel i5-6500 CPU, 8 GB DDR3 memory, without GPU and MATLAB 2012, and images in the dataset are resized to 90×150. The MCG method is the pre-trained demo from [17]. The FCN network uses a standard VGG16 structure trained by VOC2012 dataset for the feature extracting, and upsampled the outputs of the third, fourth, and seventh convolution layers. 

**Figure 15:**
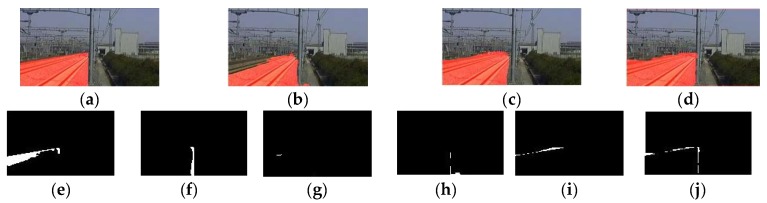
Missing and extra areas of different methods comparing with the ground truth. (**a**) Manual label of track areas. (**b**) Results of the MCG. (**c**) Results of the FCN. (**d**) Results of our method. (**e**) Missing part of MCG. (**f**) Missing part of FCN. (**g**) Missing part of our method. (**h**) Extra part of MCG. (**i**) Extra part of FCN. (**j**) Extra part of our method.

The missing part and the extra part of the segmented track area are shown in Figure 15. For the MCG algorithm, it used the CRFs to combine the fragmented regions into one unified area based on the texture which caused the missing part (as shown in Figure 15e) because of the difference texture between the nearby track and the distant track. The performances of the FCN algorithms were improved slightly from their original results in [19] because of the monotonous railway scene and the small amount of categories; but not too significantly because the shape and color textures of the scene images sampled with different illuminations, weather, and in different seasons were still complex. As shown in Figure 15f,i, the smooth boundary line of the FCN algorithm was not suitable for our railway scene parsing because of the concave and convex shapes at the straight and sharp edge of the region, especially near the area with an acute angle and straight line. Concave and convex shapes caused both a missing part and an extra part of the track area when compared with the ground truth, which would release both the missing alarms and false alarms. For the engineering application, our system would rather release a false alarm than miss a true alarm.

The performances of the three algorithms are shown in Table 4. It can be found that the proposed algorithm with four optimal Gaussian kernels achieves the highest score in PA, which means that the greatest portion of the surveillance area is found out and thus is preferred for applications.

## 6. Conclusions

The proposed algorithm uses an adaptive feature distribution extractor for railway track segmentation by making full use of the strong linear characteristics of railway scenes and the typical categories of the local areas. A good balance between segmentation precision, recognition accuracy, calculation time, and complexity of manual operation can be achieved. By using the proposed algorithm, the railway intrusion detection system can automatically and accurately delimit the boundaries of a surveillance scene in real time and greatly improve the efficiency of the system operation. Considering the fact that, in China, there are over 29,000 km of high-speed railways and the average density of cameras on high-speed railway lines is about 2.92 cameras/km, the proposed algorithm is of great significance to improve the efficiency.

The proposed algorithm can be applied into the surveillance system of public places such as airport aprons, highway pavement, and squares. These places share some common characteristics: simple structure full of straight lines—such as airplane runways and different functional areas, vehicles and different lanes, pedestrians and sidewalk lines. Before applying this method, however, the training dataset of the simplified CNN has to include new categories in such scenes, then the proposed algorithm can segment the scene and label each local area. 

## Figures and Tables

**Table 1:** Experimental results of different CNN network structures.

Kernel Size	Kernel Quantity		Calculation Time (s)	Accuracy
	C1	C2		
3 × 3	50	10	0.00372	72.25%
	70	10	0.00495	73%
	100	10	0.00689	75%
5 × 5	100	10	0.0125	76%
7 × 7	100	10	0.0217	76.5%

**Table 2:** Experiment results of different CNN network structures after the optimization.

Kernel Size	Kernel Quantity		Accuracy
	*C1*	*C2*	
3 × 3	50	10	98%
	70	10	98.5
	100	10	98.5%
5 × 5	100	10	98.75%
7 × 7	100	10	99.25%

**Table 3:** Calculation time of the comparison experiments with different workflow to segment the railway scene.

	Partial-Scanning Workflow				Complete-Processing Workflow
	Scan Time (s)	Proportion of Track Area	Segmentation and Classification Time (s)	Total (s)	Time (s)
1	0.297	41.7%	1.042	1.339	2.5
2	0.297	25%	0.625	0.922	2.5
3	0.297	75%	1.875	2.172	2.5
4	0.297	40%	1	1.927	2.5
5	0.297	30%	0.75	1.047	2.5

**Table 4:** Experimental results of different algorithms.

Algorithm		Mean IU	Mean PA	Mean EP	Time (s)
MCG		72.05%	79.94%	10.63%	7
FCN		89.83%	91.26%	16.20%	41
Our Algorithm	Four optimal Gaussian kernels	81.94%	95.90%	18.17%	0.9–2.8
	Eight regular Gaussian kernels	85.23%	93.85%	17.56%	1.1–4.4
